# Renal Nutrition and Metabolism

**DOI:** 10.3390/nu14091959

**Published:** 2022-05-07

**Authors:** Piergiorgio Messa

**Affiliations:** Unit Nephrol Dialysis & Renal Transplantat, Fdn IRCCS Ca Granda Osped Maggiore, Policlinico Milano, I-20122 Milan, Italy; piergiorgio.messa@gmail.com

Since the dawn of nephrology, dietary intervention has been one of the cornerstones of therapeutic intervention, used by nephrologists in an attempt to reduce the symptoms and metabolic complications that characterize chronic kidney disease (CKD) and, possibly, to stop its evolution. Despite this, the available evidence that clearly demonstrates these effects is still relatively few, and this evidence is contradictory and often inconclusive. For these reasons, any opportunity to stimulate the collection of new data, experiences, and opinions on this sensitive issue for nephrological culture is welcome.

The aim of the present issue of *Nutrients* is to host scientific articles that can contribute to enriching the knowledge in this field.

This collection contains ten articles of great interest, each of which explores some of the most interesting and still poorly defined aspects concerning the topic in question.

In this brief introduction, I will limit myself to providing you with a quick presentation of the topics addressed by each of the ten articles, referring to their in-depth look at the topics covered.

Silvio Borrelli and colleagues [1] look at the highly relevant issue related to the efficacy of the tools used to control the serum potassium levels that, as is well known, tend to increase with the progression of CKD, due to the reduction in the renal elimination of dietary load of potassium and other concurring factors, such as the frequent occurrence of metabolic acidosis and the widespread use of RAAS inhibitors in CKD patients. In this very well-designed observational study, the authors try to define the main variables associated with the occurrence, persistence, or resolution of hyperkalemia (HK) in a cohort of 562 CKD outpatients, observed over one year. Although the overall patients adhered to the dietary prescriptions, according to the daily urinary excretion of potassium, HK was found in almost 50% of the cohort at some time during the observation period, persisting in 17.1% overall, occurring de novo in 16.6%, and resolving in 15.6% of the patients. Among the potentially causally associated factors, the authors identified a GFR lower than 60 mL/min, the presence of diabetes, and metabolic acidosis. These data underline that HK is a frequent finding in CKD and the currently available tools for its control are only partially effective.

The handling of protein dietary intake in the different stages of CKD is another controversial issue, which was recently addressed by the Kidney Disease Outcomes Quality Initiative (K-DOQI) guidelines, which suggest restricting protein intake from the early stages of CKD. Elisa Longhitano and colleagues [2] dealt with the problems that could be encountered when implementing such a restricted protein diet in real-life medical practice. In this study, which included 220 CKD patients with a mean age of 77 years, 171 patients were eligible for a protein restriction and about 80% of them accepted, starting a diet with a restricted protein intake. An older age was the only variable characterizing the patients who refused the dietary proposal. These data, although confirming that a dietary protein restriction is well-received by most CKD patients, also stressed the need for a personalized approach to such a critical dietary prescription.

The pivotal role of microbiota changes as pathogenic factors in a large series of diseases is a topic of growing interest. However, although in the CKD field, the interest in this problem grew exponentially in the last two decades, few and contradictory data are available to date. In a paper in the present issue of *Nutrients*, I-Wen Wu and coworkers [3] report original data exploring the relationship between gut microbiota and metabolomics changes in 16 CKD patients consuming a low-protein diet (LPD-CKD), compared with 27 CKD patients who were on a normal protein dietary intake (CKD-NPD) and 34 non-CKD controls. With the limitations of the observational design of the study and the potential interference of different additional pharmacological treatments, the authors report that an LPD induced some relevant results, both positive and negative, which cast some important question marks on the possible unwanted effects of LPD, suggesting the need for new targeted experimental studies.

With the increasing number of end-stage renal disease (ESRD) patients, there was growing interest in the methods which could allow for delays in start of dialysis treatment, particularly for very elderly patients. One of the potential tools for this could be a marked reduction in protein intake; however, this could be associated with malnutrition and, hence, increased mortality rate. To counteract this potential negative outcome, many nephrologists started prescribing keto analogs (KA) as supplements. However, there is still scant evidence on whether these supplements are effective in improving the outcomes. The group of Yi-Chun Wang and colleagues [4] report data from 165 patients with advanced CKD, who were maintained with an LPD (0.6 g/kg/day) with KA supplementation, compared with 165 patients matched for the degree of CKD, who consumed an equivalent LPD without KA supplementation. The authors did not find any statistical difference in the hazard ratio for mortality between the two groups, and, even more surprisingly, KA supplementation induced a significant increase in both the risk of starting dialysis and the risk of achieving the combined outcomes (mortality or starting dialysis treatment). These interesting and very provoking results, however, need confirmation from future RCTs, including different ethnic populations.

Although kidney transplantation (KT) represents the best therapeutic option for patients who achieved the ESRD, KT recipients are still burdened by a higher risk of mortality, particularly from cardio-vascular disease, infectious and neoplastic diseases, as compared with subjects from the general population matched for age and gender. The role of different components of the diet in this clinical setting is only partially clarified. Carolien P.J. Deen and co-workers [5] face a very specific, but intriguing, topic related to niacin nutritional status after KT. In fact, the daily urinary excretion of N1-methylnicotinamide (N1-MN), a biomarker of niacin status, was associated with mortality rate in KT recipients; furthermore, it has been also demonstrated that the enzymatic conversion of N1-MN to N1-methyl-2-pyridone-5-carboxamide (2Py) is increased in KT recipients, underlining the critical importance of dosing both metabolites for drawing precise evaluation of niacin status. To gain more insights into this issue, the authors assessed the 24-h urinary excretion of N1-methylnicotinamide (N1-MN) and of its metabolite N1-methyl-2-pyridone-5-carboxamide (2Py), in 660 KT recipients, observed for a mean follow-up period of 5.4 years. The urinary excretion of N1-MN + 2Py was inversely associated with both all-cause mortality and infectious risk. These very stimulating results suggest that the correction of niacin status in RT recipients might be considered an important therapeutic target.

There is still great controversy regarding the potential benefit of a diet mainly based on foods of plant origin and/or fish-food as compared with a dietary behavior that privileges meat, dairy products and poultry in the field of CKD patients. In a very elegant study published in the present issue, Qingqing Cai and colleagues [6] evaluated the association of dietary habits and the decline of evaluated glomerular filtration rate (eGFR), using a sophisticated and very effective methodology focused on an eGFR-based dietary pattern (eGFR-DP) approach, defined on the basis of a 110-item food frequency questionnaire. The authors evaluated 78,335 participants at baseline and after 5 years. The results of this very intriguing study are somewhat controversial as compared to what is already known and are not always counterintuitive: among the most relevant results, the authors found that there are sex-specific eGFR-DPs that were independently associated with eGFR decline, different in women as compared with men, and that high protein consumption is not necessarily associated with higher eGFR decline; the variability of its effects also depends on the quality of protein used.

An increase in inflammatory markers is a quite common finding in CKD patients. Looking at the possible effects of different quality protein dietary composition on the inflammatory status in CKD, a systematic review and meta-analysis is carried out by Danielle Francesca Aycart and co-workers [7]. From the 10 controlled trial studies that met the eligibility criteria, the authors report that plant protein consumption is associated with a trend toward a decrease in C-Reactive Protein (CRP) levels, compared to animal proteins, in non-dialysis participants. However, the authors advise caution when considering the reliability of the results of these studies, as their absolute quality is relatively limited. Again, there is plenty of room for future studies of higher quality to add to the evidence on this topic.

Ran Nakamichi et al. [8] address the issue of the toxicity of elevated glucose and lipid levels on podocyte cells, whose damage, when occurs, is at the basis of the onset and, even more so, the progression of glomerular damage. In recent years, there has been an increase in diabetes due to rapid lifestyle changes, which is the main cause of CKD. The authors go through the fundamental steps in the description of the pathophysiology of podocyte damage, in the presence of the metabolic alterations that characterize some of the most frequent causes of chronic kidney disease (diabetes, metabolic syndrome). In this review, the authors combine, with admirable synthesis skills, excellent quality scientific information on the subject.

The derangements of mineral metabolism and the associated bone disease are hallmark of CKD patients (CKD-MBD). Many studies have addressed the role of protein intake in CKD-MBD progression. Much less attention has been paid to the possible role of caloric intake on this problem, which has been studied by Angela Vidal and colleagues [9] in this issue of *Nutrients*. The authors particularly focused on the interaction between caloric intake and some relevant players in CKD-MBD, namely, phosphate, FGF23 and Vitamin D, discussing the results of studies carried out either in animals or humans. In addition to the classical mineral metabolism-related pathways by which caloric intake might affect bone health, the authors also dealt with some other non-mineral mediators (adipokines, insulin, inflammation mediators, etcetera). In this review, the authors conclude that dietary caloric prescriptions in CKD patients should also consider its possible effects on mineral and bone disorders.

Kamyar Kalantar-Zadeh and coworkers [10] also deal with the unsolved problem concerning the possible beneficial effects of a predominantly plant-based diet in patients with CKD. The authors highlight the evidence supporting the beneficial effects of a patient-centered, plant-dominant, low-protein diet, which, by ameliorating the gut microbiome and modulating uremic toxin generation, might contribute to slowing CKD progression, as well as reducing cardiovascular risk, which represents the first cause of death for renal patients.

I leave you now to read these very interesting articles, and wish you a good and profitable reading.
